# A hybrid elastic-hyperelastic approach for simulating soft tactile sensors

**DOI:** 10.3389/frobt.2025.1639524

**Published:** 2025-07-22

**Authors:** Berith Atemoztli De la Cruz Sánchez, Jean-Philippe Roberge

**Affiliations:** Command and Robotics Laboratory, École de Technologie Supérieure, Montreal, QC, Canada

**Keywords:** force and tactile sensing, synthetic data, computational modeling, finite element analysis (FEA), convolutional neural networks (CNNs)

## Abstract

Efficient robotic grasping increasingly relies on artificial intelligence (AI) and tactile sensing technologies, which necessitate the acquisition of substantial data—a task that can often prove challenging. Consequently, the alternative of generating tactile data through precise and efficient simulations is becoming increasingly appealing. A significant challenge for simulating tactile sensors is balancing the trade-off between accuracy and processing time in simulation algorithms and models. To address this, we propose a hybrid approach that combines elastic and hyperelastic finite element simulations, complemented by convolutional neural networks (CNNs), to generate synthetic tactile maps of a soft capacitive tactile sensor. By leveraging a dataset of 53,400 real-world tactile maps, this methodology enables effective training, validation, and testing of each pipeline. This approach combines a fast elastic model for simple contact patches with a more detailed but slower hyperelastic model when greater precision is required. Our method automatically assesses contact patch complexity based on parameters associated with the object’s mesh to determine the most appropriate modeling technique by still ensuring accurate deformation simulation. Tested on a dataset of 12 unseen objects, our approach achieves up to 97% Structural Similarity Index Measure (SSIM) for the hyperelastic model and 90% for the elastic model. This hybrid strategy enables an adaptive balance between simulation speed and accuracy, making it suitable for generating synthetic tactile data across tasks with varying precision demands and object geometrical complexities.

## 1 Introduction

Over the past decades, various tactile sensors have been developed using diverse sensing principles, such as capacitive, piezoresistive, magnetic, piezoelectric, optical, and vision-based methods, as described in Meribout et al. (2024). A significant challenge in robotic tactile sensing lies not only in the fabrication of physical sensors but also in the design and development of algorithms that generate, process, and interpret tactile data. A common feature among the transduction techniques employed in most tactile sensors is the integration of elastomers, owing to their elastic properties that offer a soft interface for interaction. Nonetheless, the inherent complexity associated with modeling these soft materials presents substantial challenges to simulation efforts. Consequently, simulating tactile sensors for robotic grasping has emerged as a critical area of research aimed at generating accurate synthetic data that mimics the behavior of real sensors.

Due to time and resource limitations, conducting numerous physical experiments is often impractical; hence, simulations present a valuable alternative. It could significantly facilitate the creation of synthetic tactile datasets that closely replicate the properties of real data. These simulations enable testing across a wide range of scenarios, including those that may not be feasible with physical systems. While synthetic data generation has advanced significantly in the field of computer vision, greatly enhancing robots’ ability to interpret visual information, the development of other sensory modalities—particularly touch—has lagged behind. Integrating multiple sensing technologies, such as vision and tactile sensing, could enable robots to achieve more human-like perception and dexterity. Despite numerous advancements, developing tactile sensor models remains an open challenge due to the complex physics of contact interactions and the diverse principles underlying these sensors. Furthermore, accurately modeling soft interfaces requires simulation environments with advanced physics engines, which often come with high computational costs.

**FIGURE 1:**
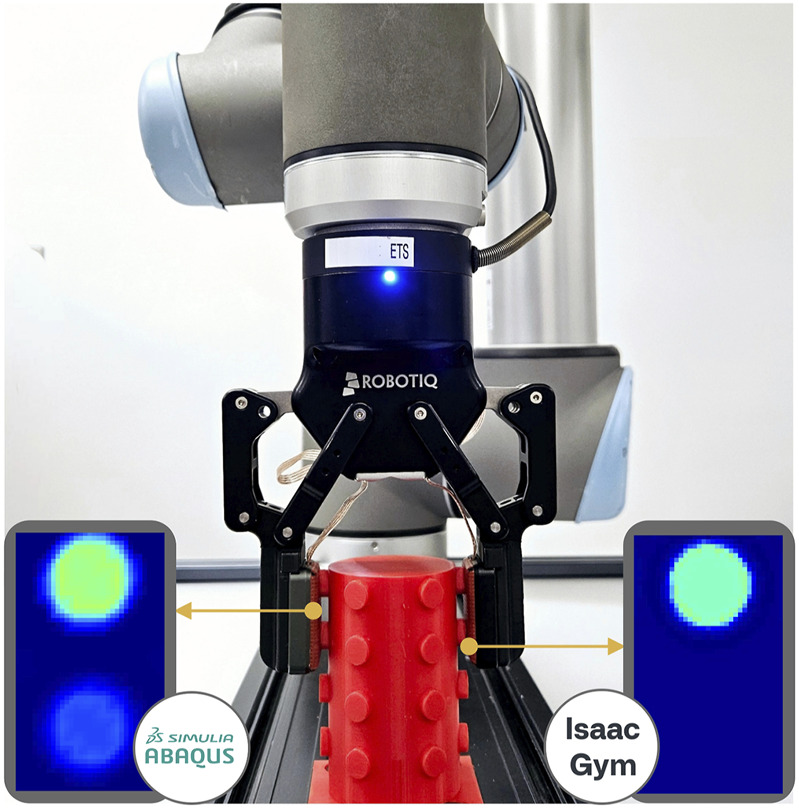
General view of the robot during a grip test. It is accompanied by comparing deformation maps between a hyperelastic model (Abaqus) and an elastic model (Isaac Gym). The cylinder has protrusions of slightly varying heights, which can occasionally cause the elastic model to miss certain details (here it is shown missing a cylindrical protrusion).

To tackle these challenges, our paper proposes a new method for simulating soft tactile sensors within an integrated robotic simulation pipeline. The pipeline is designed to enable the generation of synthetic tactile data to support future sim-to-real implementations by allowing the training and evaluation of various policies and strategies in virtual environments. In our approach, Isaac Sim serves as the foundational simulation environment. For contact interactions, we combine Isaac Gym (which uses an elastic model) with Abaqus (which employs a hyperelastic model). While the Abaqus-based approach yields more accurate results, it generally requires significantly more processing time. Conversely, simulations using Isaac Gym provide faster approximations at the cost of reduced precision, a specific case of the latter is shown in Figure 1. Additionally, a key objective of our methodology and pipeline is scalability. The constants that characterize the materials are derived from stress tests performed on material specimens. Once the simulation model is created, it can be scalable. This approach can also be applied to similar sensors that operate on capacitive principles, as they typically share a similar layered structure.

The paper is organized as follows: Section 2 provides a literature review on the simulation of tactile sensors with a focus on elastic and hyperelastic models. Section 3 describes the approach used to model a capacitive tactile sensor to generate synthetic tactile maps, utilizing an elastic model in Isaac Gym (Makoviychuk et al., 2021) and a hyperelastic model with Abaqus (Habbit Karlsson, 2013). Section 4 details the conducted experiments, the created datasets, and the validation of the simulation through comparisons between synthetic and real tactile maps across various scenarios. Finally, Section 5 summarizes the most important outcomes of the paper.

## 2 Literature review

Tactile sensor simulation is a developing field that has gained significant popularity in recent years, emerging as a crucial area for advancing robotic grasping (Li et al., 2024). While considerable progress remains to be made in this domain, one key objective is the ability to accurately capture the deformation of the sensor during contact, enabling the creation of more versatile and realistic simulations. Most reported simulations focus on rigid body dynamics, as seen in the algorithms proposed by Cremer et al. (2021), Kappassov et al. (2020), and Ding et al. (2020). However, due to the elastomeric materials used in fabrication, these approaches fail to accurately replicate tactile sensor behaviour. Alternatively, Leins et al. (2025) show that the incorporation of hydroelastic contact model into Mujoco’s physics engine can yield remarkably accurate tactile data for piezoresistive sensors, with potential extension to optical sensors. Nonetheless, scalability to larger sensor designs continues to pose a challenge.

Recognizing the need for more sophisticated modeling, researchers are increasingly adopting hybrid simulation approaches that bridge multiple software tools, thereby leveraging more powerful physics engines capable of accurately computing soft-body deformations. For example, in the SimTacLS pipeline proposed by Luu et al. (2023), a vision-based tactile sensor is simulated using Gazebo for robotic simulation and SOFA (Allard et al., 2007) for finite element analysis (FEA). In the same way, Si and Yuan (2022) rely on Ansys to calibrate their simulation model for the GelSight sensor. Another example is the integration of Isaac Gym with PyBullet, as developed by Lim et al. (2022), for implementing sim-to-real algorithms. Similarly, Wang et al. (2022) introduced Tacto, a simulator that utilizes Pyrender to simulate optical tactile sensors, combined with PyBullet for physics simulation, to generate synthetic data and train algorithms for sim-to-real algorithms. These integrated approaches, primarily used for vision-based tactile sensors, utilise two simulation tools: one for robotic simulation and another for deformation simulation. These methods rely heavily on tuning and calibration to accurately model interactions. Our methodology distinctively employs two different software tools for deformation simulation, depending on the complexity of the contact area, alongside a third tool explicitly dedicated to robotic simulation.

While hybrid simulation approaches offer comprehensive solutions for complex sensor behaviors, FEA offers precise control over mechanical properties in simulations, which is particularly useful in soft robotics. Notable examples of FEA tools include Abaqus, ANSYS, SOFA, and Mujoco (Todorov et al., 2012). The advantage of such simulations in soft robotics lies in their ability to closely mimic real-world scenarios. These simulations consider material parameters like elasticity and hyperelasticity, which are pivotal in generating more realistic data. However, the application of specialized tools like Abaqus and ANSYS in robotics remains limited due to their high computational cost, lack of real-time capabilities, and the absence of native integration with robotics frameworks, such as ROS. In the realm of simulation, IsaacSim (NVIDIA Isaac Sim, 2024) has recently gained popularity due to its photorealistic capabilities and seamless integration for training AI algorithms. Although primarily a robotic simulation software, it can accommodate basic deformations, which are merely visual renderings and may not accurately reflect real-world behavior. In this vein, some authors concentrate on simulation-based research but do not always compare their outcomes with real-world measurements. For example, TacEx (Nguyen et al., 2024), a tactile simulation framework created for the GelSight Mini sensor to support reinforcement learning, employs FEA to model sensor responses. However, its validation to date has been limited to simulation, with no reported physical testing.

NVIDIA’s Isaac Gym, functioning independently from the Omniverse simulation platform, enables soft object simulation using linear models, as shown in the work by Narang et al. (2021). In their research, the BioTac sensor is simulated using material parameters derived from an ANSYS simulation that replicates the deformations observed in the real sensor. However, these parameters may not precisely represent the real behavior of the sensor or material due to the simplification of a hyperelastic model into an elastic one. Similarly, Cui et al. (2023) use a dynamic FEM calibration approach to achieve realistic results, with the calibration parameters continuously adapting to the depth of deformation in the sensor membrane. While Isaac Gym offers several advantages, it lacks hyperelastic algorithms and only supports the implementation of linear elastic models, which fail to capture the behavior of materials such as elastomers accurately. As a result, the material parameters used in these elastic models often lack physical significance or fail to align with real-world behavior.

It is also noteworthy that most existing simulations concentrate on vision-based tactile sensors, while alternative tactile principles—such as capacitive sensors—remain relatively underexplored. Capacitive sensors offer notable versatility, as they are generally smaller than vision-based sensors, can be adapted to different sizes, and may be easier to distribute across various surfaces. For instance, Thomasson et al. (2022) adapted the sensor originally proposed by Ruth et al. (2021) to cover an Allegro Hand, illustrating the broader applicability of these tactile principles.

To address these challenges, we propose a comprehensive pipeline for generating synthetic tactile data efficiently while maintaining accuracy. Isaac Sim is used to create photorealistic environments for virtual robots, while we rely on either Isaac Gym or Abaqus to simulate the tactile sensor deformations. The selection between Isaac Gym and Abaqus is based on an estimation of contact complexity: for simpler contacts, elastic models within Isaac Gym suffice, whereas more intricate interactions requiring hyperelastic modeling are handled by Abaqus. Of the various FEA software options available for high-fidelity simulations—both commercially and in the literature—Abaqus was selected due to its robust capabilities in modeling hyperelastic materials, including its built-in Material Parameter Identification tool, which was used in this work to identify the nonlinear material properties of the sensor. On the other hand, belonging to a different category of simulation tools, Isaac Sim and Isaac Gym were chosen from the class of physics-based robotics simulators, as they support large-scale parallel simulation and seamless integration with deep learning frameworks, which are planned to be leveraged in future stages of this project. Using our pipeline, we can generate a tactile output represented by a 28-value vector, which matches the data from a real capacitive sensor with a high level of accuracy.

## 3 Proposed approach

### 3.1 Problem definition

**FIGURE 2:**
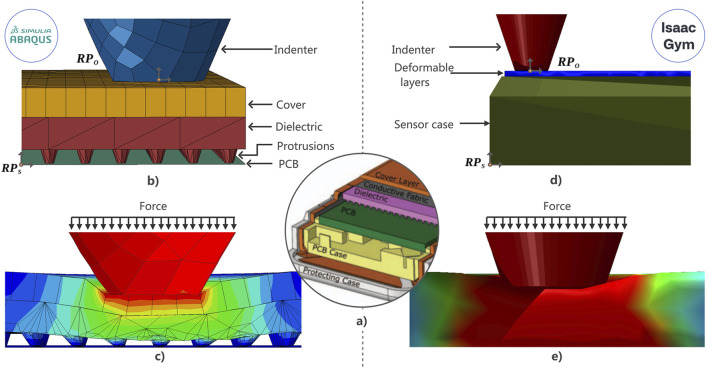
Simulations in Abaqus. **(a)** (middle of the picture) Illustration depicting the internal structure of the sensor, providing a detailed insight into its core components. **(b)** The composition of the sensor shows the different layers that constitute the simulation in Abaqus when the sensor is at rest. **(c)** Deformation of the sensor’s layers during the simulation in Abaqus when an indenter exerts pressure. **(d)** Elements of the simulation in Isaac Gym, where the deformable layer includes the neoprene cover and the dielectric of the sensor as a simplification of the model. This simplification is necessary due to a limitation of Isaac Gym, where the transmission of deformation between bodies lacks accuracy, as explained in Section 3.2.2. **(e)** Response of the deformable layer in Isaac Gym when the Indenter exerts pressure on the surface of the soft body.

The sensors involved in this work were previously reported in (Le et al., 2017; Cockbum et al., 2017). The sensor operates within a range of 0–50 N and includes an Inertial Measurement Unit (IMU), and a taxel for dynamic sensing. It has dimensions of 22 mm × 37 mm. Like other capacitive tactile sensors, a compressible dielectric is placed between the electrodes, which deforms and compresses in response to applied pressure, resulting in a change in capacitance. The internal structure of the sensor is shown in Figure 2a), where four main layers are distinguished in the following order: a printed circuit board (PCB) containing 28 taxels for static sensing, a polyurethane layer serving as the dielectric, a conductive fabric layer acting as the second plate of the capacitor, and finally, a neoprene layer that encapsulates the sensor.

One important characteristic for properly simulating the sensor is the fact that the dielectric layer contains evenly distributed conical frustum-shaped protrusions across its surface, which deform in response to applied pressure. Therefore, it is crucial to account for the shape of the dielectric in simulations, as a significant portion of the sensor’s behavior depends on the deformation of these protrusions. Such features are commonly found in the dielectrics of capacitive sensors, particularly in pillar-based designs, as they allow tuning of the compression-versus-pressure profile to achieve the desired sensing characteristics, as discussed in the review by Yuan et al. (2024).

The materials used for the dielectric and the sensor cover layer are classified as elastomers or rubber-like materials. These materials exhibit non-linear behavior, making hyperelastic models more suitable for accurately describing them. A key characteristic of these materials is their ability to undergo extremely large elastic deformations, often several times beyond their original shape. When the force causing the deformation is removed, they return to their initial state without experiencing plastic deformation. Several theoretical models have been developed to characterize this behavior, such as the Mooney-Rivlin model (Kumar and Rao, 2016), the Ogden model (Ogden and Roxburgh, 1999), and the Yeoh model (Yeoh and Fleming, 1997), all of which are based on the study of simple geometries and tests. The selection and validation of polyurethane and neoprene parameters for various hyperelastic models are discussed in detail in Section 4.1.

### 3.2 Capacitive sensor modelling

The sensor is modeled using two different simulation tools: Abaqus’s hyperelastic model and Isaac Gym’s reduction to an elastic model.

#### 3.2.1 Implementation in Abaqus

Similar to the real sensor, the Abaqus model consists of three layers. The first layer corresponds to the PCB and is simulated as a rigid shell; its mesh is composed of six three-dimensional quadrilateral rigid elements (R3D4). The second layer is a deformable solid representing the dielectric material. This layer was imported from Solidworks to accurately replicate the protuberances, and its mesh consists of 40,909 elements of the four-node linear tetrahedron type, hybrid with linear pressure (C3D4H). Finally, the last layer, modeled as a rectangular prism, is a deformable solid that simulates the sensor cover layer in conjunction with the conductive fabric layer, using C3D8RH elements for the mesh. The integration of these two layers into one is due to the markedly diminished thickness of the conductive fabric in relation to the neoprene layer. The structure of this model is depicted in Figure 2b when the sensor is at rest without deformation and Figure 2c when an object or indenter applies pressure. The nodes that are located at the edges of the layers are subjected to boundary conditions of displacement/rotation to mimic the boundary conditions by the sensor shell.

The scene in Abaqus can be practically divided into two principal components: the indenter or object that applies pressure and the sensor. Each component is associated with a reference point RP that facilitates the positioning and rotation of the body in space. The reference point $RP_{S}$ of the sensor is located on one of the corners while $RP_{O}$ for objects is located at the center of the face that serves as the base of the object. This modular setup allows the indenter to be the only element that needs updating according to the test, for instance, changing from a circular to a quadrangular indenter. The $RP_{O}$ serves as control points that enable adjustments to the position or direction of the indenter, ensuring it maintains the correct relationship with the sensor.

#### 3.2.2 Implementation in Isaac Gym

Isaac Gym operates independently from IsaacSim and uses the Flex physics engine, which only supports a particle-based approach with constraints, which allows one to simulate elastic and plastic deformations to some extent. Unlike the implementation with Abaqus, the sensor simulation in Isaac Gym involves a single layer representing all the sensor’s deformable layers, including the dielectric, conductive fabric, and cover. This simplification is necessary because the software does not accurately simulate the transmission of deformation from one layer to another when superimposing soft bodies, which causes the simulation to fail with certain geometries. In this case, the simulated sensor includes a casing that encloses the main sensor, its PCB, and a deformable soft layer (see Figure 2d). The effect of the indenter deforming the soft layer is then illustrated in Figure 2e. The sensor’s reference point $\left(RP_{S}\right)$ is positioned in the upper right corner, and the contacting object’s reference point $\left(RP_{O}\right)$ is located at the center of mass to make the test object interchangeable within the scene. The algorithm presented by Hu et al. (2020) is implemented to create a mesh with 30,993 tetrahedral elements for the soft object representing the deformable layers of the sensor. Given that this simulation involves elastic material models, the Poisson’s ratio and Young’s modulus were determined by approximating values from deformation data obtained through the stress tests described in Section 4.1.

Isaac Gym’s versatility allows integration with the UR10 robot, Robotiq 2-Finger gripper, and tactile sensors. This simulation environment is particularly valuable for scenarios involving the coordination between robot trajectory planning and tactile sensing.

### 3.3 Overview of the entire data generation pipeline

**FIGURE 3:**
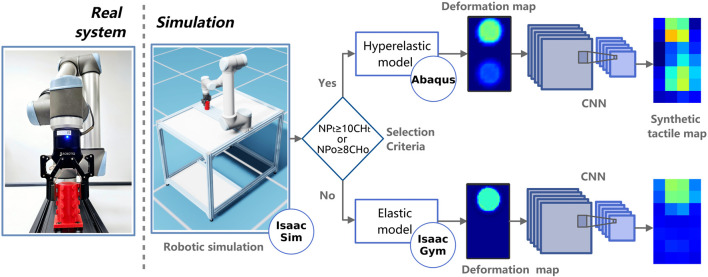
An overview of the proposed pipeline. The sensors are simulated and coupled to the gripper and the robot, and subsequently, the selection criteria dictates whether to use an elastic or hyperelastic model. These models generate deformation maps, critical for producing the final synthetic tactile maps.

The system is implemented using Isaac Sim in conjunction with Isaac Gym and Abaqus, controlled *via* Python scripts, as depicted in Figure 3. The primary scene, referred to as the “main scenario,” is developed in Isaac Sim. This scenario encompasses the robot pedestal, the UR10 robot, and the Robotiq 2F-85 gripper, which is equipped with a tactile sensor on the fingers, serving as the end effector. The object of interaction is also included in this scenario. The design allows the pipeline to alternate between hyperelastic and elastic models for generating synthetic tactile maps, based on the characteristics of the contact. In particular, the choice of using elastic (generally less accurate but faster) or hyperelastic (generally more accurate but slower) is determined by analyzing the contact area between the sensor and the object using a decision process further described in Section 3.3.1. An accompanying video further illustrates and explains this approach, providing a dynamic visual representation of the system’s functionality.

The simulation progresses as the robot moves from its initial position to a vertical grip position relative to the object, as illustrated in Figure 1. When the gripper attains the specific grasping coordinates, the fingers begin to close until a collision is detected between the sensor and the object. This event activates a deformation simulation process analysis using one of the selected elasticity models, which incorporates data regarding the object’s position, sensor coordinates, and the applied force. For the purposes of this study, it is assumed that all objects are completely rigid and that their geometrical properties are known.

In both Abaqus and Isaac Gym, the simulations produce a 33 × 57 data matrix that represents the deformation of the sensor surface nodes. We process this matrix as an image, referred to as a deformation map, which is used as an input to a convolutional neural network (CNN) which determines the simulated tactile sensor’s output. The CNNs are crucial in this context as they map the deformation input to a taxel output, effectively learning the complex electromechanical relationships between the deformation map and the tactile map.

#### 3.3.1 Selection criteria (Isaac or Abaqus)

One of the key differences between elastic and hyperelastic models is how they describe the deformation of soft bodies under applied forces. The hyperelastic model more accurately reproduces the real behavior of dielectrics, capturing finer details and reducing simulation errors compared to the more rigid elastic model, particularly as contact complexity increases. Based on the reconstruction of the object’s mesh from its STL file, we identified four parameters that are often related to the complexity of the sensor’s contact area with the object. These parameters are: the total number of points in the object’s mesh (NPt); the number of unique edges extracted from the convex hull’s triangle mesh over the entire object (CHt); the number of points in the sensor’s overlapping area on the object (NPo); and the number of unique edges extracted from the convex hull’s triangle mesh in the overlapping region (CHo).

**FIGURE 4:**
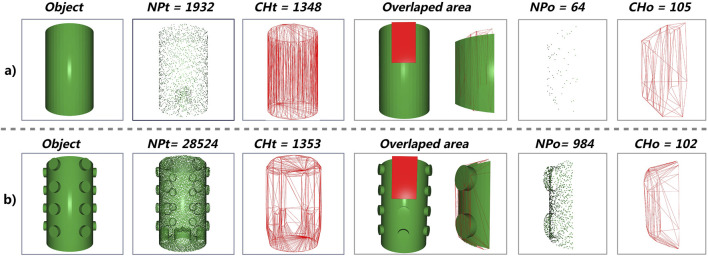
Comparison of two objects illustrating the selection criteria. **(a)** Flat object without reliefs. **(b)** Object with reliefs or more details. For each object, the columns from left to right show: the object itself, the point cloud describing the object (NPt), the number of unique edges derived from the triangulated convex hull of the full point cloud (CHt), and the sensor interaction area. The sensor interaction area includes the overlapped point cloud (NPo) and the number of unique edges derived from the triangulated convex hull of that region (CHo).

There is a significant relationship between these parameters. An object with more intricate details requires a higher NPt, as more points are needed to define the triangles that form its three-dimensional geometry. The convex hull, which is the smallest convex shape that completely encloses the object, serves as the basis for another parameter: the number of unique edges derived from its triangulated surface. This edge-based descriptor does not vary drastically with the level of detail in the object, as it is primarily influenced by the object’s global shape. As shown in Figure 4, for both objects, the number of unique edges remains similar—both for the whole object (CHt) and for the sensor interaction area (CHo)—whereas NPt and NPo change significantly as surface complexity increases.

Empirical results from our experiments indicate that for objects with detailed geometry, the NPt value is at least 10 times the CHt value and the NPo value is at least 8 times the CHo value—necessitating a hyperelastic simulation with Abaqus, since the elastic model is generally not able to accurately simulate these fine details. Figure 4 illustrates the relationship between two distinct examples. In the first example (a), a flat cylinder produces ratios of approximately NPt≈1.43CHt and NPo≈0.6CHo . In contrast, the more detailed cylinder in case (b) reveals significantly higher ratios, with NPt≈21CHt and NPo≈9.6CHo . The differences in implementation and the outcomes of both models are elaborated upon in Section 4.2.

#### 3.3.2 Generation of tactile maps

To bridge the sim-to-real gap and identify the underlying function that maps simulated deformations to real tactile maps, separate CNNs has been trained for each pipeline: one for the elastic pipeline and another for the hyperelastic one. For training each of the networks, the same dataset was used, consisting of 13,000 deformation maps from a real-world dataset created for this project, as reported by De la Cruz-Sánchez et al. (2025). For each test, the sensor is fixed to a base and pressure is applied with different interchangeable indenters adapted to the tip of a Mark-10 dynamometer. A Mitutoyo Metric Dial Indicator, adapted to the Mark-10’s base, is employed to measure the indentation depth throughout the testing process. Forty-nine different indenters were used, including basic geometric shapes such as triangles, squares, spheres, and circles in various sizes. The primary rationale behind using a variety of irregular indenters was to engage all the sensor taxels in multiple ways, thereby producing a wider variety of tactile maps. For each test, the sensor response is obtained in tactile counts (TC) for the variation in force in newtons (N) applied by the indenter. These tests were performed in a force range of 0–50 N.

Abaqus and Isaac Gym simulations generate deformation matrices where each pressure map has dimensions of 33×57 × 1. Given the nature of the problem, each deformation and tactile map is processed as an image, enabling the use of convolutional neural networks (CNNs) as a suitable approach. A dataset of 13,000 tactile maps was shuffled and split into an 80/20 ratio for training and validation. This split was consistently applied to both CNNs across each pipeline.

The elastic and hyperelastic modeling pipelines employ an identical CNN architecture, identified through Keras Tuner’s Hyperband tuning algorithm. An exhaustive search demonstrated that the structure and values of the layers, filters, and neurons consistently converged within a similar range for both Abaqus and Isaac Gym simulations. To ensure consistency and enable a fair comparison between the two simulation approaches, we selected the hyperparameters from one of the best outcomes of the tuning process and applied them to both networks. The final structure consists of three convolutional layers with filter sizes of ( 2×2 , 80), ( 2×2 , 95), and ( 2×2 , 120), respectively. Each convolutional layer is followed by a max-pooling (MaxPool) layer, with all pooling layers employing 2×2 max-pooling with a stride of one. The resulting feature map is then flattened and connected to a fully connected layer containing 570 neurons, activated by ReLU. This layer is subsequently linked to 28 linearly activated neurons, representing the output and predictions for each real sensor taxel. To evaluate the network, a custom loss function is employed that computes the sum of absolute differences between the predicted and true sensor outputs, effectively quantifying the overall error across the output dimensions, as expressed mathematically by Equation 1. Where N denotes the number of elements in the output vector.

$$L\left(y_{\text{true}},y_{\text{pred}}\right)=\overset{N}{\underset{i=1}{\sum }}\left|y_{\text{true},i}-y_{\text{pred},i}\right|$$ (1)

## 4 Experiments

### 4.1 Material characterization and validation

The material constants in the equations of hyperelastic models are obtained from experimental tests. This is done to obtain the most accurate constants possible for the neoprene and polyurethane used in the sensor. Although it is possible to find these parameters in the literature, these values are general, and the material of each manufacturer may have small variations. To determine the mechanical properties of polyurethane and neoprene, stress tests are performed on standardized specimens based on ISO 3167 type A specifications (ISO, 2014).

Subsequently, the stress-strain data obtained for each material are imported into the Abaqus/Explicit software for evaluation and to obtain the coefficients for different constitutive models. Of the variety of models available in Abaqus, the Ogden and Mooney-Rivlin models were the only ones that presented stability and no inconsistencies during the parameter acquisition process. The Ogden model is a general model expressed in terms of the principal applied stretches and where $\lambda_{1}$ , $\lambda_{2}$ and $\lambda_{3}$ are the stretch ratios, $\alpha_{k}$ and $\mu_{k}$ are material parameters based on the experimental data (Bergström, 2015). The model is extendable up to N order, incorporating a term D as an incompressible parameter to account for volume consistency under deformation. The representation used by Abaqus is shown in Equation 2:

$$\Psi \left(\lambda_{1}^{*},\lambda_{2}^{*},\lambda_{3}^{*}\right)=\overset{N}{\underset{k=1}{\sum }}\frac{2\mu_{k}}{\alpha_{k}^{2}}\left(\lambda_{1}^{*\alpha_{k}}+\lambda_{2}^{*\alpha_{k}}+\lambda_{3}^{*\alpha_{k}}-3\right)+\overset{N}{\underset{k=1}{\sum }}\frac{1}{D_{k}}{\left(J-1\right)}^{2k}.$$ (2)

While the Mooney-Rivlin model uses the strain energy density function U , where $C_{10}$ and $C_{01}$ are constants for each material (Youssef, 2022). Their representation in Abaqus is given by Equation 3:

$$U=C_{10}\left({\bar{I}}_{1}-3\right)+C_{01}\left({\bar{I}}_{2}-3\right)+\frac{1}{D_{1}}{\left(J-1\right)}^{2}.$$ (3)

The material parameters obtained for both models by Abaqus/Explicit are shown in Table 1.

**TABLE 1:** Material coefficients for hyperelastic models.

Material	Model	Parameters	RMSE (mm^2^)
Neoprene	Ogden	$\alpha_{1}=8.74091$	0.0082
		$\mu_{1}=0.0027$	
		$\alpha_{2}=0.3889$	
		$\mu_{2}=0.0551$	
	Mooney-Rivlin	$C_{10}=0.1786$	0.0077
		$C_{01}=0.0022$	
Polyuréthane	Ogden	$\alpha_{1}=1.4538$	0.0080
		$\mu_{1}=0.1351$	
		$\alpha_{2}=-4.9910$	
		$\mu_{2}=0.2223$	
	Mooney-Rivlin	$C_{10}=0.1706$	0.0091
		$C_{01}=0.0040$	

Another important factor to consider in the performance of the simulation is its ability to reproduce the behavior of the dielectric material. The sensor in this work has evenly distributed conical frustum protrusions throughout the layer. Each cone has a height of 0.5 mm, a base diameter of 0.6 mm, and a tip diameter of 1.2 mm, with a distance of 1.2 mm between each protrusion. Therefore, to evaluate the parameters of the models presented in Table 1, we asses the deformation of the cones under a normal applied load.

**FIGURE 5:**
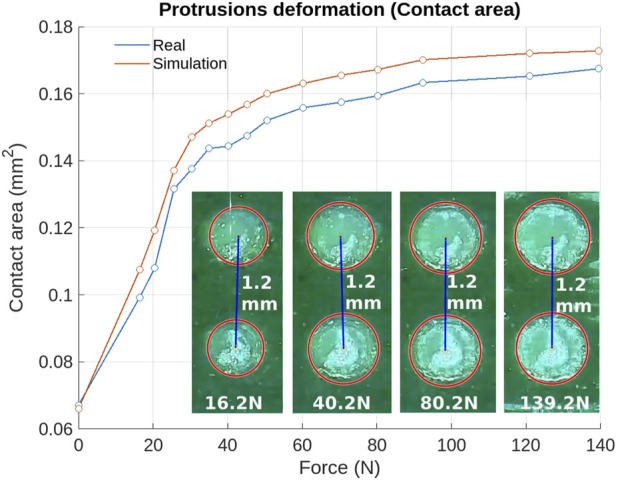
Validation of the material characterization using FTIR theory through measuring the protuberances’ contact area with the dielectric deformation under an applied normal force. The increase in the contact area of the dielectric cones is highlighted in a red circle in the images below the deformation curve.

For this evaluation, a 22 × 37 mm dielectric sample underwent compression tests to assess cone deformation using the frustrated total internal reflection (FTIR) theory. The test setup included a camera positioned beneath an acrylic base, which was illuminated by LEDs, with the dielectric sample placed on top. The phenomenon of total internal reflection enabled the camera to detect the contact areas of the cones, which varied according to the normal force applied. The images were then analyzed with Matlab by detecting the circles encompassing the dielectric contact area in each image. This monitoring was done over time in relation to the applied normal force and as shown in Figure 5. As greater force was applied, the contact area increased, but not necessarily linearly. The same tests were simulated in Abaqus using the corresponding material parameters. The Root Mean Squared Error (RMSE) between the real and simulated data for the contact area was calculated to be 0.008 $mm^{2}$ , confirming that the hyperelastic model accurately describes the behavior of the dielectric layer. Although the validation was performed in a range of 0–140 N, the force range in which the gripper operates, where the physical sensor is mounted, does not exceed 50 N.

The stress-strain data from the stress tests previously conducted for the hyperelastic models were utilized to determine the parameters for the elastic model, specifically Young’s modulus and Poisson’s ratio. Young’s modulus, which represents the slope of the stress-strain relationship, was calculated to be 1.289 MPa. Additionally, Poisson’s ratio, which indicates the ratio of transverse strain to longitudinal strain, was determined to be 0.1729.

### 4.2 Results: comparison of synthetic tactile maps vs. real

For all evaluations between synthetic and real tactile maps, the Structural Similarity Index Measure (SSIM) was used as a performance metric and will be handled throughout the results presented in this document. The SSIM index measures the similarity between two images, yielding a value between 0 and 1, where one indicates that the images are identical and 0 indicates that the images are completely different. Unlike pixel-by-pixel comparison methods such as Mean Square Error (MSE) (Wang and Bovik, 2009), SSIM assesses perceived visual quality by accounting for changes in structural information. This makes it particularly well-suited for evaluating spatial patterns and textures, as it considers luminance distortion (l(x,y)) , contrast distortion (c(x,y)) , and loss of correlation (s(x,y)) (Wang et al., 2004). These factors are expressed in Equation 4, which corresponds to the factorized form of the SSIM equation:

$$SSIM\left(x,y\right)={\left[l\left(x,y\right)\right]}^{\varphi }\cdot {\left[c\left(x,y\right)\right]}^{\omega }\cdot {\left[s\left(x,y\right)\right]}^{\gamma }.$$ (4)

Where φ,ω , and γ control the weight of each component in the SSIM score and are typically set to 1.

Since tactile maps are ultimately treated as images, it is more relevant to evaluate the structural similarity rather than isolated taxel values. Contact deformations typically excite multiple neighbouring taxels, resulting in spatial patterns across the sensor. For this reason, SSIM was chosen to evaluate the performance of the CNNs trained for each simulator, ensuring that they could generalize their performance effectively to unseen data. We divided the testing stage of the trained networks into two phases. The first phase consists of 400 unseen real-world test cases using different indenters. For the Abaqus CNN, an SSIM of 0.81 was obtained for the first test dataset and 0.82 for the Isaac CNN (compared to 0.87 and 0.86 previously obtained on the validation dataset, respectively).

The second phase of testing involved evaluating the performance of the CNNs within the entire pipeline, with the goal of observing their generalization capabilities in a real-world work environment. Therefore, the tactile sensor was integrated into a Robotiq 2F-85 parallel gripper to interact with more realistic shapes observed in robotic grasping tasks. The tests, comprising 2,000 real-world trials, focused on the gripping of 12 different everyday rigid objects. Each trial captures the sensor’s response from the onset of the gripper’s closure until the object is securely held.

To investigate the CNNs’ behavior in the presence of sensor hysteresis, 50 trials per gripper-object position were conducted with the physical sensor, with a force of 50 N applied in each test. Indeed, despite consistent test conditions and constant excitation patterns, variability in taxel tactile counts can still be observed, attributable to the inherent properties of the real sensor. This phenomenon has been previously examined by Kwiatkowski et al. (2022). To address this issue, the tactile data are compared with deterministic synthetic tactile maps, which remain constant under identical simulation conditions. This comparative analysis—between a single synthetic map and a set of real tactile maps obtained under the same conditions—is essential for evaluating the generalizability of the synthetic tactile maps produced by both pipelines.

**FIGURE 6:**
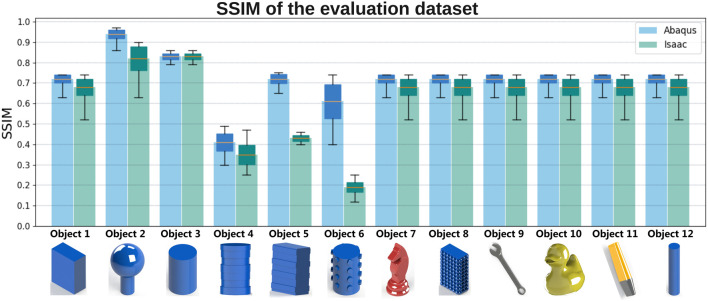
SSIM of the validation dataset for both simulation pipelines. Blue bars represent the average SSIM for each object obtained using Abaqus, while green bars represent those obtained using Isaac Sim. Each bar graph also displays the distribution of SSIM values, illustrating the variability of the results across trials.

The results obtained using the SSIM for the second testing dataset across each of the pipelines are shown in Figure 6, where the average SSIM for each object is shown, as well as box plots to compare the distribution and trend of the results obtained. Overall, for the 2,000 tests, an average SSIM of 0.72 was achieved for Abaqus and 0.63 for Isaac Gym. Furthermore, all synthetic tactile maps generated in this study are available in a comprehensive dataset (De la Cruz-Sánchez et al., 2025). This dataset includes both the physical sensor test data and the corresponding synthetic tactile maps produced by the Abaqus and Isaac pipelines, offering a valuable resource for further analysis and validation.

**FIGURE 7:**
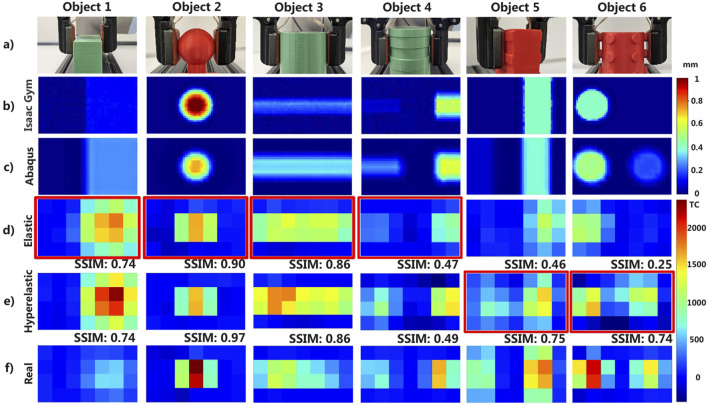
Comparison of synthetic tactile maps from simulations with elastic and hyperelastic models for six basic objects. **(a)** Real 3D printed and simulated objects. **(b)** Deformed dielectric surface in the simulation of the object and robot in Isaac Gym. **(c)** Deformed dielectric surface in the simulation of the object and robot in Abaqus using a hyperelastic model. **(d)** Synthetic tactile map obtained from Isaac Gym using an elastic model. **(e)** Synthetic tactile map obtained from Abaqus using a hyperelastic model. **(f)** Real tactile map obtained from the actual sensor. For better visualization, the tactile maps were rotated 90° counterclockwise from the sensor’s original vertical position, as shown in Figure 1. The synthetic tactile maps of rows **(d,e)** have been highlighted with a red border, which would be the tactile maps generated at the end of the pipeline considering the selection criteria step.

Of the 12 rigid objects used to create the dataset, 6 were selected for further investigations and to illustrate some relevant and distinctive cases for each of the studied approaches. These include two rectangular prisms, three circular prisms, and one sphere. These objects represent a diverse range of bodies, with some having completely smooth surfaces while others feature more intricate structures. This selection highlights six key scenarios that may arise in practical applications, offering a valuable opportunity to evaluate the limitations of each developed pipeline. The corresponding results are presented in Figure 7, where each column represents a different object, and the rows show the outputs from the Abaqus and Isaac Gym simulations, along with the synthetic and real tactile maps. The deformation map is in mm and uses the Jet colour scheme, where red and darker tones indicate greater deformation and blue signifies no deformation. Similarly, synthetic and real tactile maps use blue for a tactile count of 0 and dark red for a tactile count of 2,400.

For smooth objects, such as rectangular and circular prisms as well as the sphere, the synthetic tactile maps exhibit lower errors compared to those obtained from more detailed objects. Specifically, for objects 1, 2, and 3, both Isaac and Abaqus synthetic tactile maps show an excitation pattern relatively similar to the real one. The deformation maps produced by Abaqus show that, even across the best and worst test cases, SSIM values remain close to the dataset average, indicating a stable and relatively uniform performance with minimal deviation from the mean. Isaac Gym, in parallel, exhibits greater variation in those extreme cases yet still maintains robust overall performance.

In contrast, objects 4, 5, and 6, which contain more detailed structures, present two distinct cases. For object 4, both pipelines successfully reproduce the deformation caused by the object’s reliefs, resulting in a similar SSIM value. However, for objects 5 and 6, the height differences between local features vary by 0.5 mm, and in such cases, the elastic simulation is not accurate enough to replicate the real deformation pattern. It incorrectly assumes that the sensor has lost contact with one of the reliefs, whereas this is not observed in the real tactile maps. This is a clear example of how an elastic simulation, combined with a simplified sensor model, can result in a tactile map where an entire feature is missing.

In general, as the level of detail increases, the elastic simulation begins to show a higher level of inconsistency, capturing fewer details compared to the hyperelastic model. This comparison is illustrated in Figures 7b,c, where the level of detail captured by the deformation maps is crucial for creating accurate synthetic tactile maps. These results align with initial expectations based on the selection criteria outlined in Section 3.3.1, to reinforce this, Table 2 shows the 4 parameters that are evaluated to decide between an elastic or hyperelastic pipeline for the 6 objects reported in Figure 6. In this figure, the synthetic tactile maps generated by the selected pipeline are highlighted in red, emphasizing the models that best meet these criteria. Objects with a ratio of ten times the number of points in the mesh (NPt) compared to the unique edges (CHt) of the object exhibit more detailed deformation patterns. Both simulations yield similar tactile excitation patterns for objects with reliefs, such as objects 2 and 4. However, in the figure, differences in the tactile count values are noticeable through the colour intensity of each taxel. While these variations are noticeable at the local level, the SSIM metric provides a global measure of similarity across the entire tactile map. In future practical implementations, generating accurate excitation patterns is more relevant than determining the exact value of individual taxels.

**TABLE 2:** Selection criteria parameters for 6 objects.

Object	Selection criteria				Pipeline
	NPt	CHt	NPo	CHo	
1	1,356	231	51	36	Isaac Gym
2	13,830	15,537	1,205	3,444	Isaac Gym
3	1,932	1,348	64	105	Isaac Gym
4	5,472	1,449	164	141	Isaac Gym
5	5,628	426	242	90	Abaqus
6	28,524	1,353	984	102	Abaqus

Another important parameter to monitor is the time it takes for the Abaqus and Isaac Gym pipelines to generate a tactile map. Table 3 presents processing time metrics for creating a dataset of 2000 synthetic tactile maps, replicating the real-world testing of the 12-object gripping test dataset. In some cases, the generation time for a tactile map in Isaac Gym can reach 157.6 s, which is four times the average, whereas in Abaqus, this time can be up to 11 times the average. Despite the larger range in Isaac Gym, with a ratio of maximum to minimum times of 64, compared to 38 in Abaqus. Notably, the maximum time in Isaac Gym approximates the average time in Abaqus, highlighting significant variability in processing times between the two systems.

**TABLE 3:** Processing time for Abaqus and Isaac Gym.

Simulation	Abaqus	Issac
Average (s)	162.1	37.4
Minimum (s)	48	2.46
Maximum (s)	1863	157.6
Standard deviation (s)	110.9	32.44

## 5 Conclusion

In this study, we developed two simulation pipelines for a soft capacitive tactile sensor. One pipeline relies on an elastic model, while the other is based on a hyperelastic model. Both pipelines use FEA theory and a CNN to create synthetic tactile maps. In cases where the elastic simulation fails to capture object details, the resulting tactile map may exhibit a completely different pattern than the real map. In such situations, the hyperelastic model is more suitable, albeit at the cost of increased computation time. For example, according to Table 3, generally, simulations with Abaqus take 3.5 times longer than those with Isaac Gym, but in complex cases, the difference can be up to 11 times. However, when the contact area is simple, both pipelines generally produce similar patterns in the tactile map. In these cases, using the elastic model is a viable option, as it allows for faster computation with Isaac Gym.

The results from the presented cases indicate that both pipelines can generate viable synthetic tactile maps. The choice between them depends on the required accuracy and the time available for the simulation. Therefore, we propose a selection criteria grounded in empirically defined thresholds, enabling users to adjust the simulation parameters according to each task’s specific requirements. Although the hyperelastic pipeline exhibited superior accuracy, it is essential to evaluate the trade-offs between speed and accuracy when constructing datasets for real-world applications. To illustrate a potential use case, in insertion tasks involving pegs with simple geometries, the elastic model may suffice; however, more complex surface features may demand the higher fidelity of the hyperelastic pipeline.

While the current object set focuses on representative cases, it provides a valuable foundation for demonstrating the pipeline’s capabilities. It serves as a basis for future expansion toward more diverse geometries. Furthermore, the pipeline is currently designed for rigid objects and considers access to 3D mesh data, which presents an opportunity for future work involving deformable objects and more uncertain or partial object representations. Future work will focus on validating the practical utility of the synthetic tactile data in real-world tasks, moving beyond similarity-based comparisons. In the long term, we aim to develop a workflow that utilizes extensive simulated data to train AI algorithms for direct deployment on physical robots. To foster further research and collaboration, we have made our complete dataset publicly available in the TactileDataset repository (https://github.com/Lab-CORO/TactileDataset). This repository includes all physical sensor tests along with the corresponding synthetic tactile maps generated using both the Abaqus and Isaac pipelines. As the project evolves, new tests and object data will be regularly added.

## Data Availability

The data supporting the results of this study are available in our GitHub repository at TactileDataset (https://github.com/Lab-CORO/TactileDataset).
